# Book Reviews

**Published:** 1895-09

**Authors:** 


### Book Reviews.

TWENTIETH CENTURY PRACTICE. An International Encyclopedia of Modern Medical Science. By leading authorities of Europe and America. Edited by THOMAS L. STEDMAN, M. D., New York City. In twenty volumes. Volume III. Occupation Diseases, Drug Habits and Poison. New York: William Wood & Co. 1895.

These volumes are following each other with a rapidity that is commendable and in keeping with the usual promptitude of the celebrated publishers. This volume opens with an article by Norman Kerr, of London, entitled Alcoholism and Drug Habits, which covers the first 137 pages of the book. This eminent author is quite competent to deal with this many-sided subject, and he has done so in this instance with much elaboration and great satisfaction. The insidiousness with which alcohol, opium, cocaine and other inebriating substances creep through the gates and alleys of the body, demands alertness on the part of the physician to detect their destructive influences while yet in their incipiency and before they have done permanent harm, and Dr. Kerr here points out the evils and the way to meet them. He divides inebriate phenomena into two great groups of morbid symptoms. One consists of the toxicating effects produced on body and brain by the injurious influence of the poisonous articles on tissue, organ and function. The other comprises the diseased conditions which, in whole or part, operate in certain individuals to impel them to "take to drink" or to give themselves up to other forms of narcotic or anesthetic indulgence. We have stated the foregoing in the author's own words because its phraseology could not be condensed or changed without doing violence to its clearness.

In dealing with narcomania, which Kerr terms the disease of inebriety, the author furnishes us with a new and expressive term which signifies a mania for intoxication or any intoxicant. This is a great improvement over the term dipsomania which is a misnomer and leads to confusion.

The next subject dealt with is shock and collapse of which George F. Shrady, of New York, is the author. The literature of medicine is constantly increasing in this direction, and we are glad to find a section set apart to its consideration in this volume; but we submit that this article might have been considerably extended with profit. Eugene Boise, of Grand Rapids, has recorded interesting observations that are not referred to in the bibliography or the text.

There is no subject of more interest to the average *voyageur* than seasickness, or naupathia, to which the next section of this work is devoted. The author, Albert L. Gihon, of the U. S. Navy, was a fit person to select to write on this subject since his experience, both on sea and land, for the past forty years is a ripe one from which to draw conclusions. There are many points in this essay that ought to be understood by the general public, and we think that it would not be out of place to abstract it and publish it as a magazine article. As soon as travelers can be made to understand that seasickness is a neurosis and not a stomach disorder, *per se*, great progress will be made in its prevention. Dr. Gihon says that in his personal experience of forty years in the naval service and on board numerous passenger vessels, on every sea and during some of the most terrible hurricanes and typhoons ever encountered, he has never met with a fatal case, nor one in which death might probably result. Further on Dr. Gihon remarks—we quote *verbatim :* "Nevertheless, something may be done in the way of precaution. The ocean traveler usually prepares for a sea voyage as a bride is gotten ready for marriage, by weeks of anticipatory sur-excitement and bodily fatigue ; and just as the young girl starts on a tour in a feverish, asthenic condition demanding rest, quiet and care for its reparation, and wholly unfitted for the performance of those new functions which involve still greater excitement and expenditure of vital force, so does the housewife, exhausted by onerous domestic preparations for departure, after a long period of sewing, shopping, packing and farewell visiting, involving reckless exposure, loss of sleep and irregular meals, undertake to encounter physical environments that may be such as to demand the utmost constitutional vigor. It is little more surprising, therefore, that the offspring of the one should bear the marks of parental infirmity than that the other should become actually and seriously ill. Other imprudents, both men and women, who have been spared physical exhaustion, gorge themselves at the last moment with confectionery or disorder their digestion by injudicious indulgences, which increase their susceptibility to seasickness beyond the power of bromides, choloform or strychnine to overcome." Dr. Gihon then proceeds to explode the theory that seasickness is a good thing to "clear out the system"—a popular notion with nine-tenths of inexperienced ocean travelers. Dr. Gihon has befittingly served the cause of humanity in this essay.

The next section is devoted to a consideration of mountain sickness and is written by Georg von Liebeg, Munich. It is a fit complement to the preceding paper, and should be carefully studie by climatologists and physicians who are consulted with reference to changes of climate or environment for patients.

Osteomalacia is treated of in a short essay by W. T. Councilman, of Boston, and then follow two papers by Dr. Gihon, one on heat-stroke and the other on frost-bite. Dr. Gihon's rhetoric is charming and his papers always prove entertaining and instructive.

We now come to the section devoted to a consideration of the diseases of occupations, by James Hendrie Lloyd, of Philadelphia. This paper shows the chemical, mechanical and atmospheric effect of occupations on the economy and deals with overwork and nervetire as a result. It is a valuable contribution and merits careful reading.

Lastly, the section on poisoning is reached, which is well prepared by Beaumont Small, of Ottawa, and James Stewart, of Montreal. The usual well-arranged index closes this volume.

A System of Surgery. By American Authors. Edited by Frederic S. Dennis, M. D., Professor of the Principles and Practice of Surgery, Bellevue Hospital Medical College, New York; President of the American Surgical Association, etc., assisted by John S. Billings, M. D., LL. D., D. C. L., Deputy Surgeon-General, U. S. A. To be completed in four imperial octavo volumes, containing about 900 pages each, with index. Profusely illustrated with figures in colors and in black. Volume I., 870 pages, 422 engravings and two colored plates. Price, per volume, $6,00 in cloth; $7.00 in leather; $8.50 in half morocco, gilt back and top. Full circular free to any address on application to the publishers. Philadelphia: Lea Brothers & Co. 1895.

It affords us genuine satisfaction to place this book in a convenient place on our library table. There is a wholesome "fitness of things" in presenting a work on surgery entirely written by American authors. We take this occasion to congratulate the editor and publishers on the parts they are playing respectively in this enterprise. There is no reason why American surgeons, who are confessedly among the foremost in the world, should not place their stamp upon the literature of surgery in a distinctly national fashion. It is perfectly absurd for Americans to be imbued with the belief that to learn surgery they must go abroad and the present work will do much to neutralise that tendency.

The first section of this book, consisting of 144 pages, is devoted to a consideration of the history and literature of surgery. The work proper opens with a section on surgical pathology, including inflammation and repair of wounds, to which 100 pages are devoted. Then follow a section on general bacteriology of surgical infection, to which about ninety pages are allotted. The succeeding chapter, comprising about fifty pages, considers symptoms, diagnosis and treatment of inflammation, abscess, ulcer and gangrene. A chapter of thirty pages is devoted to septicemia, pyemia and poisoned wounds. Traumatic fever, erysipelas and tetanus get brief consideration in the next eighteen pages. Rabies, hydrophobia and lyssa receive treatment in the next twelve pages. A chapter, consisting of seventy pages, is set apart to the consideration of gun-shot wounds. Fractures and dislocations are elaborately dealt with in the next 130 pages. The important subject of anesthesia occupies thirty pages. The technique of antiseptic and aseptic surgery is considered in the next fifty pages. The final section, consisting of 135 pages, is set apart to the consideration of operative surgery. A very complete index of twenty-five pages closes the book.

The following is the list of authors in Volume I.: Herman M. Biggs, John S. Billings, William H. Carmalt, Phineas S. Conner, William T. Councilman, Frederic S. Dennis, A. G. Gerster, Charles B. Vancrede, Stephen Smith, J. Collins Warren, William H. Welch and Horatio C. Wood.

The scope and import of this volume can be understood by the foregoing synopsis. We cannot enter into details, nor is it necessary. This promises to be the best surgical treatise that has been issued by any press or under the direction of any editor up to date. The first volume is of a character to justify this opinion.

TRANSACTIONS OF THE AMERICAN ASSOCIATION OF OBSTETRICIANS AND GYNECOLOGISTS. Volume III., for the year 1894. Octavo, pp. 530. Price, cloth, $5.00 ; half Russia, $6.00. Philadelphia : Wm. J. Dornan, Printer, 1895.

This association annually puts forth a volume that ought to be the pride of every member of the organization. It is our privilege to have access to the annual transactions of most of the national special societies, and, without disparagement to any other, we feel warranted in asserting that there is no superior work done, nor is it published in any better form than by this association.

The book before us, bound in half-Russia with red edges, is decidedly the handsomest volume of society transactions that it has been our privilege to look upon in many a day. Moreover, in turning its pages, its contents proves to be of the highest standard in quality. The papers, for the most part, are upon subjects that are yet more or less in dispute as to essentials in pathology, practice or technique; while the discussions are charmingly vigorous, impersonal and instructive.

A careful examination of the book reveals the work of a marvelously well-disciplined,society, consisting of a hundred or more earnest men that are doing a valuable service in the cause of humanity. Membership in such a body is surely an honor that its Fellows ought to bear with dignity and pride, and that ought to stimulate the ambition of every specialist in obstetrics, gynecology and abdominal surgery in America.

The papers herein published have appeared in the special journals, either in full or in abstract, hence need not be referred to here in detail. The volume is handsomely and somewhat profusely illustrated.

Transactions of the Southern Surgical and Gynecological Association. Volume VII. Edited by W. E. B. Davis, M. D., Secretary. Published by the Association. Philadelphia: William J. Dornan. 1895.

This book contains the papers read at the seventh session of the association, held at Charleston, S. C., November 13, 14, 15, 1894. It is uniform in style and make-up with the preceding volumes.

Year by year these books, as they are issued, mark the progress of the specialties with which they deal in that portion of the Republic which we technically speak of as the South. There is no more creditable work done by any association of medical men and its published transactions are a credit to its distinguished secretary, whose wisdom in founding the association is accentuated more and more as time advances.

